# Supplementary material for: Seminoma and Embryonal Carcinoma Footprints Identified by Analysis of Integrated Genome-Wide Epigenetic and Expression Profiles of Germ Cell Cancer Cell Lines
Source: PLoS One. 2014 Jun 2;9(6):e98330. doi: 10.1371/journal.pone.0098330 (PMC4041891; doi:10.1371/journal.pone.0098330)
Supplement: File S1 — ZIP file containing DMRforPairs output for significant regions. Please start from the html files. (ZIP) [file pone.0098330.s008.zip › figures/34332.pdf]

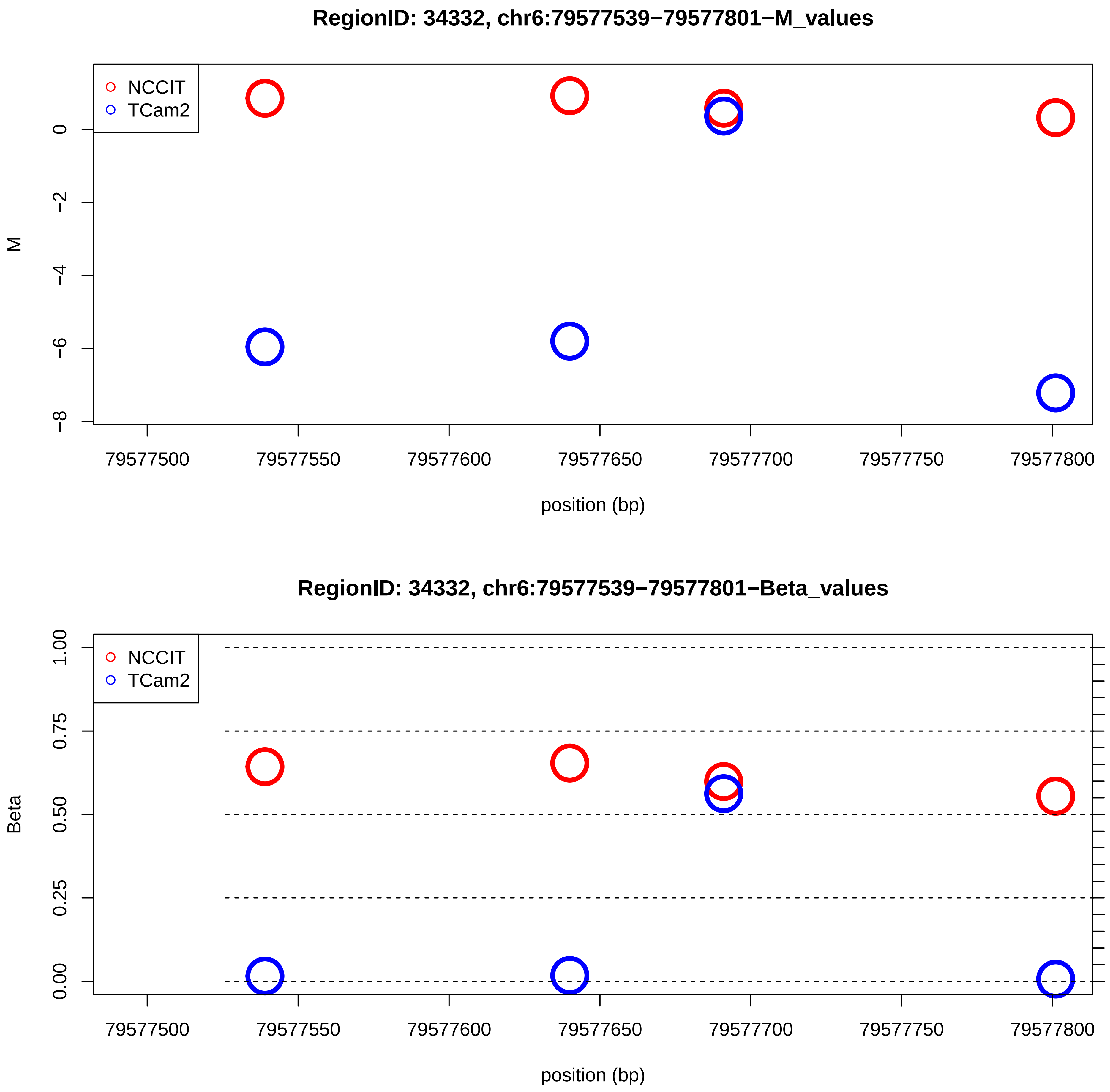
RegionID: 34332, chr6:79577539−79577801−M_values
NCCIT
TCam2
M
0
−2
−4
−6
−8
79577500
79577550
79577600
79577650
79577700
79577750
79577800
position (bp)
RegionID: 34332, chr6:79577539−79577801−Beta_values
NCCIT
TCam2
Beta
1.00
0.75
0.50
0.25
0.00
79577500
79577550
79577600
79577650
79577700
79577750
79577800
position (bp)
